# Comparing pronunciation challenges in South Korean preschoolers with unilateral single-sided deafness due to cochlear nerve deficiency to a norm-referenced standard

**DOI:** 10.1371/journal.pone.0297640

**Published:** 2024-02-23

**Authors:** Goun Choe, Jong Woo Lim, Hyun Jung Lee, Seung Hyun Kim, Marge Carandang, Bong Jik Kim, Byung Yoon Choi

**Affiliations:** 1 Department of Otolaryngology-Head and Neck Surgery, Chungnam National University Sejong Hospital, Chungnam National University College of Medicine, Sejong, Republic of Korea; 2 Department of Otorhinolaryngology, Seoul National University Bundang Hospital, Seongnam, Republic of Korea; 3 Department of Otorhinolaryngology-Head and Neck Surgery, Tondo Medical Center, Metro Manila, Philippines; 4 Sensory Organ Research Institute, Seoul National University Medical Research Center, Seoul, Korea; ITALY

## Abstract

This study aimed to compare the development of pronunciation in South Korean preschoolers with unilateral cochlear nerve deficiency (CND) to that of age-matched preschoolers with normal hearing, a topic that has not been explored previously. In a retrospective analysis, 25 preschoolers with unilateral CND who had undergone a speech evaluation battery, including a pronunciation and vocabulary test, were enrolled. Utilizing the Urimal Test of Articulation and Phonation and customized language ability tests, pronunciation and vocabulary were assessed. The subjects’ speech evaluation scores were converted into age-adjusted z-scores using normal controls’ data. While vocabulary performance was within normal limits, their average pronunciation z-score was -2.90, significantly lower than both the zero reference point and their vocabulary z-scores. None of the subjects scored above average in pronunciation. Thirteen patients were recommended for articulation therapy, seven were considered as potential candidates for this therapy, and the remaining five were within normal limits. There was no observed correlation between the development of pronunciation and vocabulary. Notably, some subjects’ pronunciation scores did not improve, even after serial follow-up during their preschool years. Despite typical vocabulary development, preschoolers with unilateral CND exhibit significant delays in pronunciation. These findings emphasize the necessity for vigilant monitoring of their language development.

## Introduction

Cochlear nerve deficiency (CND) is a prevalent cause of congenital unilateral sensorineural hearing loss, accounting for 25.8–43.7% of all cases [1, 2]. In the past, it was widely believed that unilateral hearing loss did not significantly impact a child’s language development [3]. However, recent research challenges this notion, suggesting that children with unilateral hearing loss may experience educational or behavioral issues, coupled with lower speech-language scores [4–8]. These challenges could stem from the difficulties associated with hearing in noisy environments and localizing sounds, which are typical for individuals with single-sided hearing.

Nonetheless, children with unilateral CND often receive less clinical attention post-diagnosis compared to those with other types of hearing loss. This disparity could be attributed to the perceived complexity of treating or rehabilitating their hearing, or the mistaken belief that they can hear without problems due to their normal hearing in one ear. Regrettably, these patients often do not receive ongoing clinical evaluations, even though unilateral CND can be identified through universal newborn hearing screening.

Language development, which includes proficient pronunciation, is crucial for effective speech communication in daily interactions. While previous studies have explored academic achievement and vocabulary development in children with unilateral CND and single-sided deafness (SSD), pronunciation has not been a focus of research. Furthermore, given the diverse etiologies of unilateral hearing loss, analyzing the pronunciation of the unilateral CND cohort exclusively could help to isolate the effects of other potential causes.

This study aims to fill this gap by investigating the pronunciation development of preschoolers with unilateral CND. We hypothesized that these children would exhibit poorer pronunciation skills compared to typical hearing peers. Our goal is to assess their pronunciation development and juxtapose it with their vocabulary growth, aiming to ascertain potential delays in the pronunciation development when compared to the established developmental benchmarks in prior research. Through this research, we seek to provide practical insights for clinical practice and contribute valuable information to academic discussions in this area.

## Materials and methods

### Ethics statement

This study was approved by the Institutional Review Board (IRB) of Seoul National University Bundang Hospital (SNUBH, Seongnam, Republic of Korea) and adhered to the principles of the Declaration of Helsinki (IRB No: B-2212-798-107). Given the retrospective nature of the study, it was impractical to obtain consent due to the long observation period or completed follow-ups. Furthermore, there were no reasons for subjects to refuse consent, and the IRB of SNUBH waived the requirement for obtaining informed consent, considering there were no potential harms. The data was accessed for research purposes between November 28, 2022, and March 23, 2023. The authors had access to information that could identify individual participants only during data collection and no further access after collection.

### Subjects

In this study, data were collected retrospectively from all preschoolers diagnosed with SSD due to unilateral CND at tertiary care hospitals between November 2018 and May 2022. We defined preschoolers as children aged between 24 and 83 months, and included all who met the study criteria. The SSD criteria were based on the auditory brainstem response threshold (ABRT) test, which necessitated a response to a sound stimulus of 25 dB or lower in the normal ear, and either no response or a response exceeding 90 dB in the deaf ear. If the ABRT test was not conducted, we used pure tone audiometry data as a substitute. We included cases where the average thresholds at 0.5, 1.0, 2.0, and 4.0 kHz were less than 25 dB in normal ears and more than 81 dB in deaf ears. Unilateral CND was diagnosed based on internal auditory meatus (IAM) magnetic resonance imaging (MRI). All of our SSD subjects due to CND had IAM nerve grades of I (only one nerve visible in the IAM), II (two nerves visible in the IAM), or III (three nerves visible in the IAM), according to the previously proposed IAM grading system [9]. We only included children who had never used hearing aids or other hearing assistance technology. All subjects were healthy children with no reported comorbidities, developmental delays, anomalies, cognitive or motor impairments, or history of ear infections or surgeries. We excluded children with any of the aforementioned disabilities that could potentially affect their hearing ability.

Initially, 32 children were enrolled. At our research institute, it is standard practice to administer a speech evaluation battery, which includes a pronunciation test, to all children with SSD, including those with unilateral CND. However, seven of these children did not undergo the pronunciation test due to various reasons, such as lack of cooperation or insufficiently developed language skills for testing. Consequently, we included data from 25 children in the study. Among these 25 subjects, 16 were boys, and the average age was 45.04 months (range, 24–82 months). We analyzed the data from subjects’ speech evaluation tests based on the results of their most recent visit. The data for each subject are presented in S1 Table.

### Speech evaluation

#### Evaluation of pronunciation

The Urimal Test of Articulation and Phonation (U-TAP) was employed to assess pronunciation capabilities. U-TAP, an articulation and phonation test, is extensively utilized in clinical settings for children in South Korea [10–12]. It was developed based on norms established by Korean linguists who analyzed the pronunciation of typically developing children aged between 2 years and 0 months to 6 years and 11 months. These analyses were conducted in the standard language regions of Seoul and Gyeonggi. The pronunciation of each age group was evaluated and scored to determine the average and standard deviation values. These results were subsequently used to formulate U-TAP, offering a reliable and valid approach for assessing children’s pronunciation abilities in accordance with age-specific standards. U-TAP evaluates the accuracy of consonants and vowels at both the word and sentence levels. During the U-TAP test, subjects must complete a picture-naming task that comprises 30 items. They are prompted to articulate 1–3 sentences containing specific keywords while observing 9 images. The examiner then assesses the accuracy rates of 43 consonants and 10 vowels. The determination of the level of articulation therapy required for children is predicated on the accuracy rate of the consonants.

While the basic U-TAP test encompasses 43 consonants and 10 vowels, our institute has traditionally prioritized consonants in pronunciation evaluations, especially when time is constrained. This is because consonants are considered more critical than vowels in determining the necessity for articulation therapy. Consequently, in this study, we examined the accuracy rates of consonants in the U-TAP and transformed raw scores into z-scores to categorize the level of pronunciation development. The U-TAP test is divided into four categories based on consonant accuracy: 1) recommended for articulation therapy (z≤-2), 2) considered for articulation therapy (-2<z≤-1), 3) within normal limits (-1<z≤0), and 4) above average (z>0).

#### Evaluation of vocabulary

The primary instrument utilized for assessing vocabulary skills was the Receptive and Expressive Vocabulary Test (REVT) [13]. The REVT is a test specifically designed to gauge the levels of vocabulary development across various age groups. This test was formulated by assessing the vocabulary skills of healthy adults and children, ranging from 2 to 18 years old, residing in Seoul and Gyeonggi Province, who do not exhibit any learning difficulties. The data gathered from these assessments were then used as the standard norms for evaluation. In instances where a child’s level of language development posed a challenge to the administration of the REVT, we opted to use the Sequenced Language Scale for Infants (SELSI) as a substitute [14]. The SELSI has been developed to assess the receptive and expressive language abilities of 4 to 35 months through parental reports. To ascertain the age-appropriate level of vocabulary development, the raw scores were transformed into age-adjusted z-scores.

#### Statistical analysis

All statistical analyses were conducted using SPSS for Windows, version 25 (IBM Corporation, Armonk, NY, USA), and the data were visualized with GraphPad Prism 7.00 (GraphPad Software, California, USA). The results are expressed as means ± standard deviation (SD). The z-score was determined by subtracting the mean score from the individual raw test score and then dividing the result by the SD value. The Shapiro-Wilk test was used to assess the normality of the data, while the Mann-Whitney U-test, one-sample Wilcoxon test, Wilcoxon matched-pairs signed rank test, and Spearman correlation analysis were used to analyze the clinical variables. A *p*-value of less than 0.05 was considered statistically significant.

## Results

### Significantly poorer pronunciation in children with unilateral CND than in normal controls

**Fig 1:**
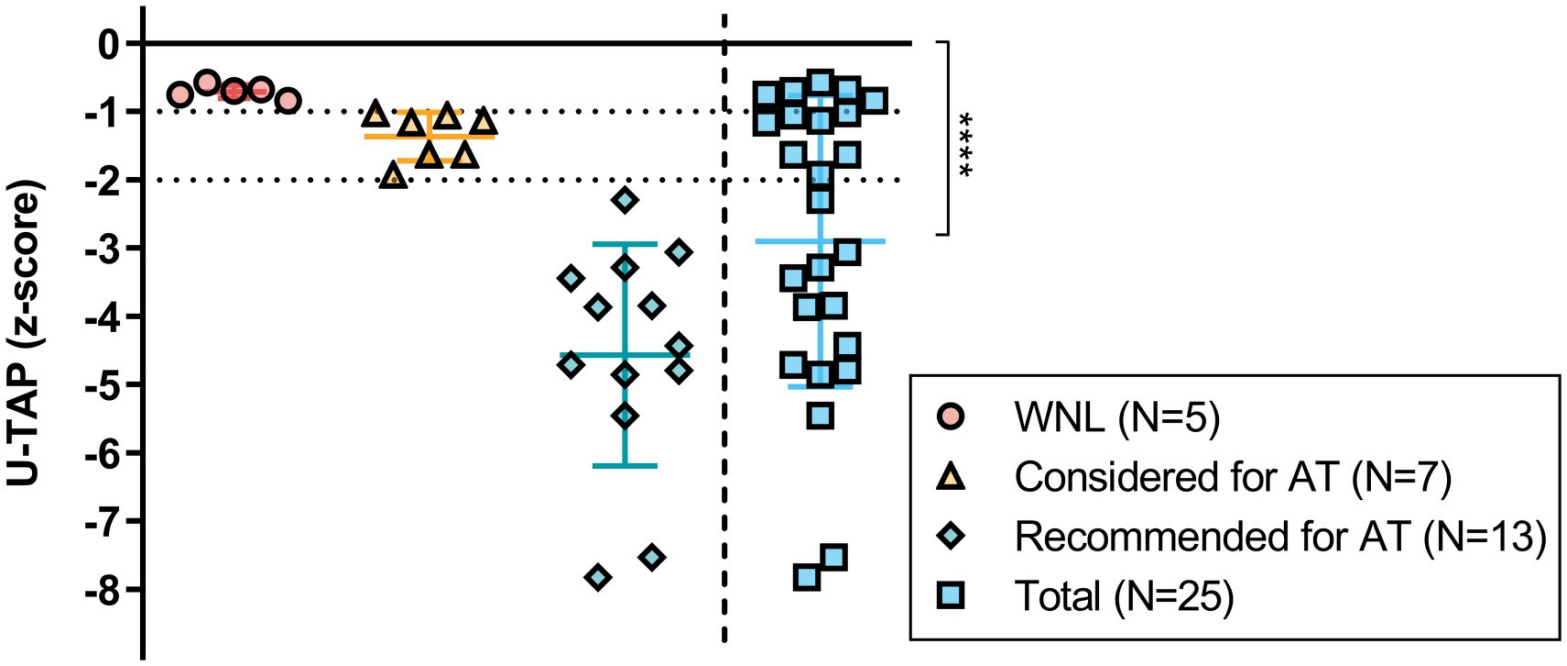
U-TAP z-scores of 25 children with unilateral cochlear nerve deficiency. WNL, within normal limits; AT, articulation therapy; ****p<0.0001.

The U-TAP z-scores for all 25 subjects fell below 0, with an average of -2.90 ± 2.13, exhibiting a non-normal distribution (*p* = 0.011, Shapiro-Wilk test). This z-score was significantly lower (*p*<0.0001) than the standard reference point of 0 (Fig 1). Given that U-TAP is a test for evaluating pronunciation accuracy, it can be inferred that the pronunciation of the 25 children with unilateral CND involved in this study was significantly inferior to that of their typically developing age-matched peers.

When the subjects were categorized based on the need for articulation therapy, 80% (20/25) of the children were identified as candidates for this therapy (Fig 1). Specifically, thirteen subjects were recommended for articulation therapy, while it was suggested that the remaining seven subjects should at least consider it. The pronunciation accuracy of the remaining five subjects fell within normal limits.

Spearman correlation analysis was conducted to examine the relationship between age (in months) and the z-score of U-TAP. The analysis revealed a weak linear relationship, as evidenced by a correlation coefficient of -0.26. The *p*-value was non-significant at 0.19, suggesting that there was no significant correlation between age and pronunciation.

### Lack of significant improvement in pronunciation accuracy in children with unilateral CND despite serial follow-up during the study period

**Fig 2:**
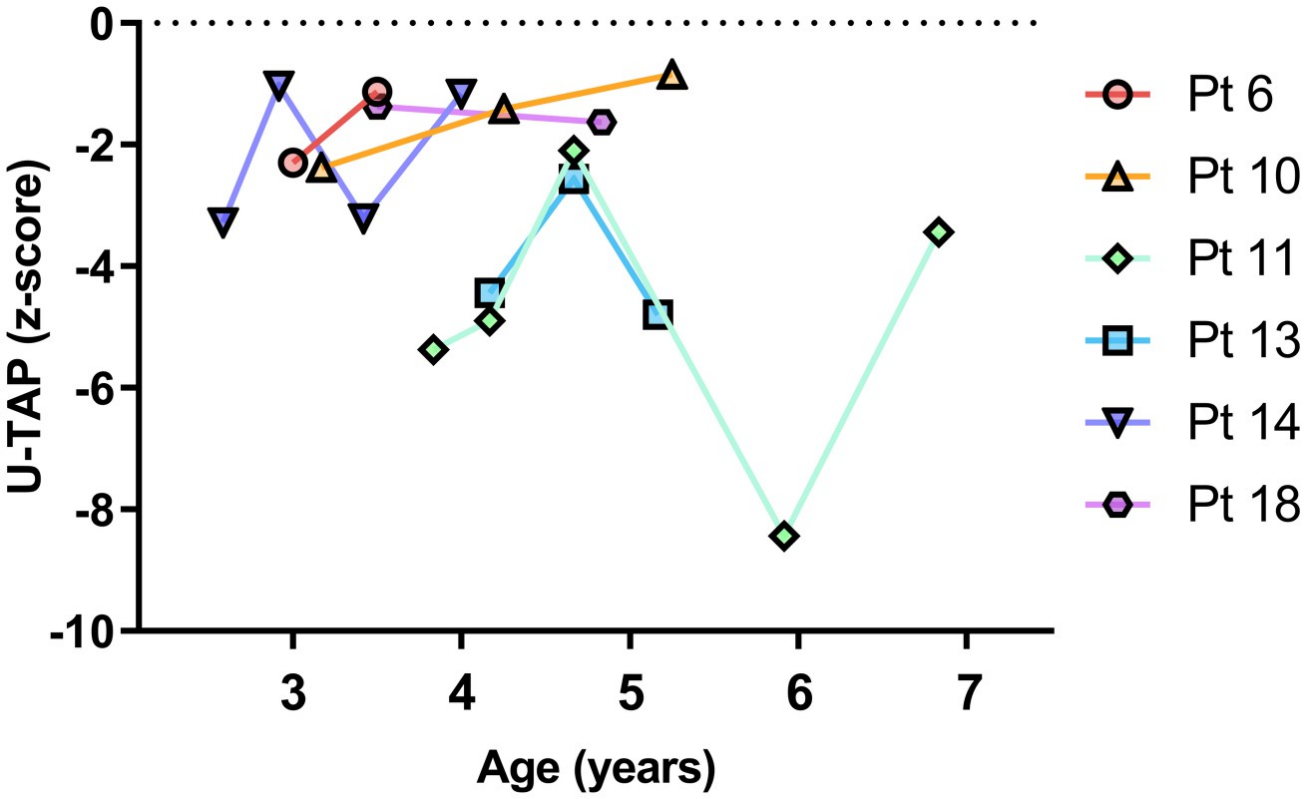
Serial U-TAP z-scores based on age.

Out of the 25 subjects, six children underwent serial regular speech evaluation tests. Their serial U-TAP z-scores are presented in Fig 2. Despite the passage of time, their age-adjusted z-scores for pronunciation accuracy did not improve over time, except for patient 10. Moreover, there was no significant correlation between their age and z-score (*p* = 0.36).

### Differential development between vocabulary and pronunciation accuracy

Out of the 25 subjects, 23 completed the vocabulary assessment. The REVT was substituted with the SELSI in 3 subjects due to their level of language development. Their receptive vocabulary raw scores were converted to age-adjusted z-scores. The average z-score was 0.03±1.38, which is normally distributed and shows no significant difference when compared to the normal reference point of 0.

**Fig 3:**
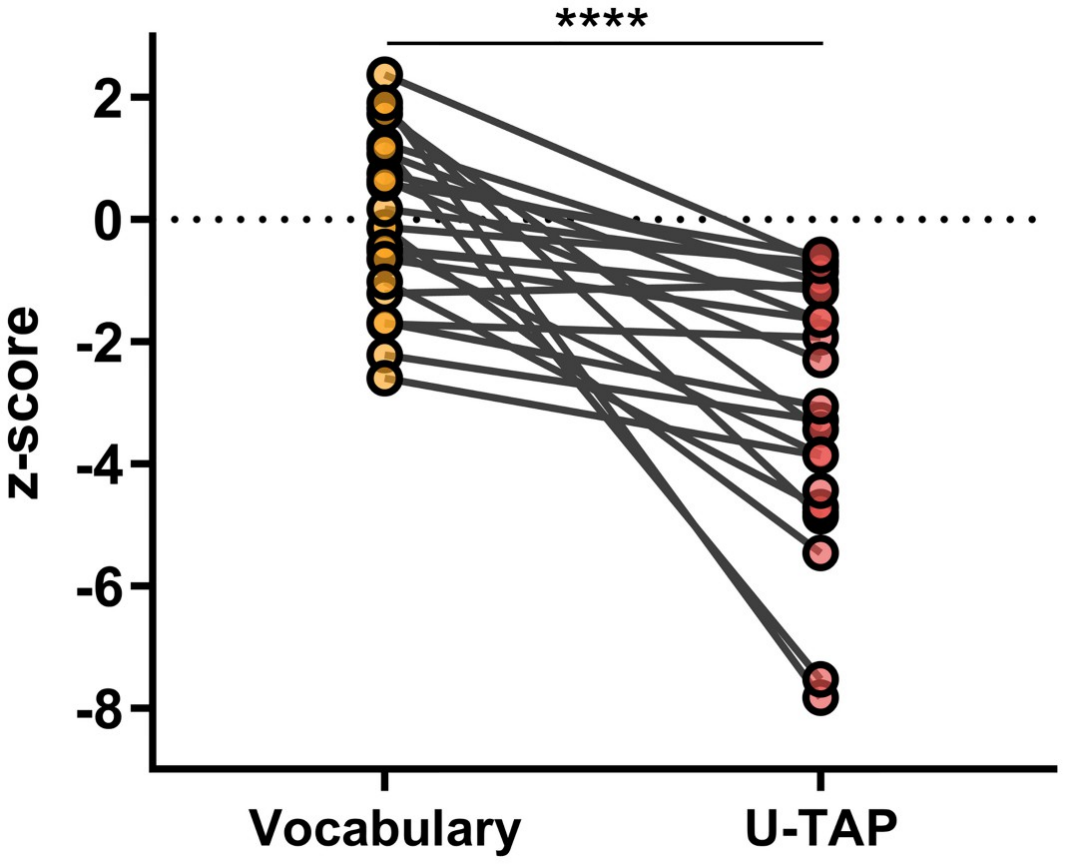
Paired z-scores of receptive vocabulary performance and pronunciation performance (U-TAP). ****p<0.0001.

While the receptive vocabulary scores stayed within the normal range, the subjects’ pronunciation scores, at -2.90 ± 2.13, were significantly below average. Importantly, a significant difference was observed when conducting a pairwise comparison of the z-scores between the vocabulary test and the U-TAP (*p*<0.0001) (Fig 3).

### Lack of correlation between pronunciation accuracy and vocabulary scores in children with unilateral CND

**Fig 4:**
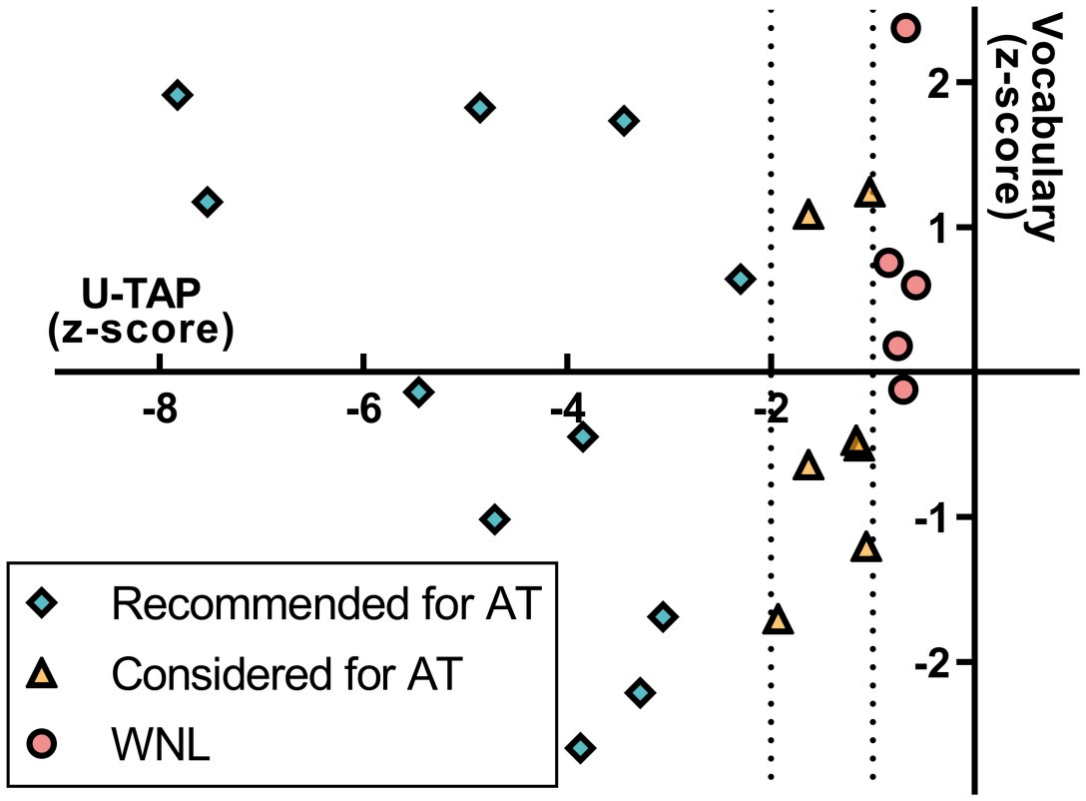
Correlation of U-TAP and vocabulary z-scores. The blue, yellow, and red circles on the graph represent three groups divided based on the need for articulation therapy. The x-axis and y-axis depict the z-scores of U-TAP and vocabulary, respectively.

We investigated whether there was any correlation between the U-TAP and vocabulary test scores for the 23 preschoolers with unilateral CND who underwent both tests. As illustrated in Fig 4, no correlation was found between the two scores (R^2^ = 0.012, *p* = 0.83).

### Delayed vocabulary development in one-third of preschoolers with unilateral CND and pronunciation problems

**Fig 5:**
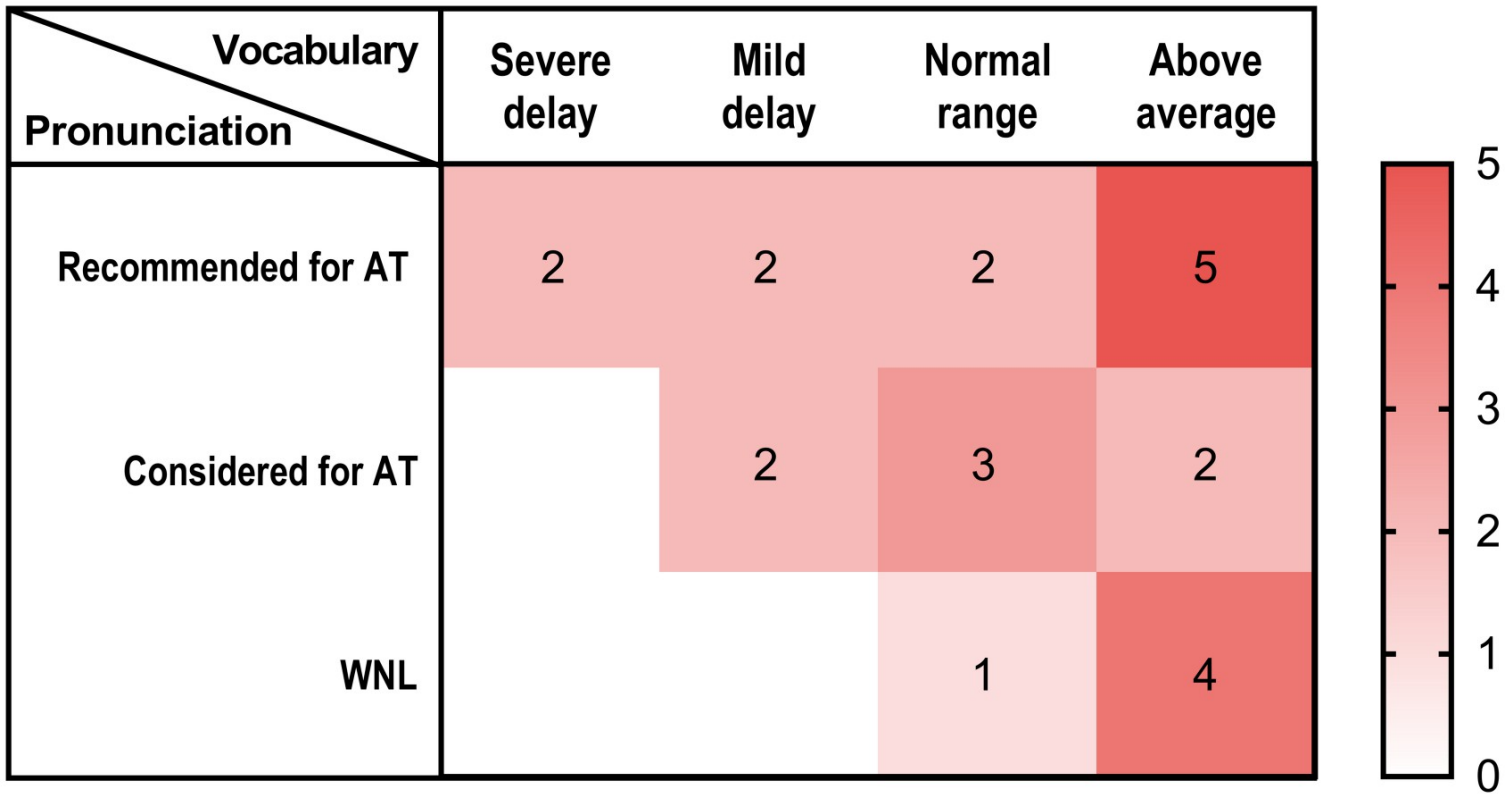
The number of subjects according to the degree of pronunciation and vocabulary development. AT, articulation therapy; WNL, within normal limits.

Although no statistically significant correlation was identified between pronunciation scores and vocabulary scores, a descriptive trend was noted in the clinical classification system. This trend suggested that individuals with lower graded pronunciation often had a higher proportion of delayed graded vocabularies (Fig 5). Among the 11 subjects with poor pronunciation, as determined by U-TAP test results, who were recommended for articulation therapy, there was a wide range of vocabulary skill development, from severe delay to above average. Of these 11 subjects, four (36.4%) exhibited a noticeable delay in vocabulary abilities. Among the seven subjects who may require articulation therapy, only two (28.6%) children demonstrated a mild delay in vocabulary skills. In total, six (33.3%) of the 18 subjects with pronunciation issues showed a significant delay in vocabulary abilities. Conversely, the vocabulary abilities of the five preschoolers, whose pronunciation was within normal limits, all fell within the normal range or were above average.

## Discussion

Previous research has demonstrated that unilateral congenital hearing loss can have numerous adverse effects. Children with SSD often exhibit poorer spatial hearing and lower word recognition scores compared to their peers with normal hearing, as they do not benefit from binaural hearing [15, 16]. Asymmetry in auditory input also affects how the brain processes information [17–19]. Children with SSD have been found to have lower IQs than their peers with normal hearing [20, 21]. They also demonstrate a deficiency in complex verbal working memory tasks, particularly in processing unfamiliar verbal information, and maintaining verbal information while processing irrelevant verbal information [22]. Behavioral and linguistic deficits are observed in children with SSD beginning in infancy [23–25], and their development of grammatical skills is often inadequate. Studies have also reported lower scores on receptive and expressive language examinations in these children [4, 26]. These impairments can negatively affect their behavior and academic performance [4, 27, 28], and may lead to social and emotional problems.

Pronunciation accuracy in preschoolers with congenital SSD has not been previously described, largely because it has not been the primary focus of speech-language development research in this population. Furthermore, the significant etiologic heterogeneity of SSD complicates the task of confirming whether the SSD is indeed congenital, thus preventing a definitive conclusion about the impact on speech development in cases of congenital SSD. With this in mind, our study aimed to investigate potential sequelae on vocabulary development and pronunciation accuracy in these subjects, with a specific focus on CND, a cause of SSD that is definitively congenital.

In this study, 25 preschoolers with unilateral CND demonstrated significantly lower average pronunciation scores compared to their typically developing, age-matched peers. Most of these children either required or were considered for articulation therapy to address their delayed pronunciation accuracy. Interestingly, despite their substantial difficulties with pronunciation, their receptive vocabulary performance was generally within or near the normal range, albeit with some individual variations. This suggests that the children with unilateral CND had significantly poorer pronunciation relative to their receptive vocabulary.

Preschoolers with SSD due to CND who showed normal or near-normal pronunciation were highly likely to have normal receptive vocabulary development. However, this was not always the case for children with unilateral CND, and no significant correlation between receptive vocabulary performance and pronunciation scores was observed. Despite this, it was found that if the pronunciation z-score was within the normal range or better than -1, the vocabulary performance was also within the normal range or above average. Nonetheless, among the 18 subjects with a z-score of -1 or worse, a third exhibited delayed receptive vocabulary scores. This implies that preschoolers with unilateral CND and pronunciation difficulties may require additional evaluation to identify potential delays in vocabulary development.

Cross-sectional studies have demonstrated a delay in speech development among preschoolers with unilateral hearing loss. However, it is crucial to ascertain whether this delay continues as children mature, which can be achieved through longitudinal follow-up studies. Previous literature has reported that the receptive language and verbal intelligence of preschool-age children with unilateral hearing loss lagged behind their peers with normal hearing, but showed significant improvement upon entering school [29]. In contrast, our study of a unilateral CND cohort did not reveal any notable enhancement in the relative pronunciation level over time among children who underwent serial follow-up tests. Despite one out of five children showing potential improvement visually, we were unable to statistically confirm this. This underscores the importance of closely monitoring the pronunciation accuracy of children with unilateral CND. Furthermore, given that all subjects in our study were preschoolers with an average age of 45 months, we advocate for consistent, long-term speech evaluation follow-ups for school-aged children with unilateral CND. This will help determine whether their delayed language development improves with age, or with exposure to articulation therapy or schooling.

Most individuals with unilateral CND live without special interventions post-diagnosis, which can result in their language development being easily overlooked or neglected. However, our study discovered that these individuals often exhibit below-average pronunciation skills during their preschool years. In cases where children demonstrate significantly poor pronunciation accuracy, and where articulation therapy is considered or recommended, a third of these children also exhibit a delay in receptive vocabulary. Consequently, we recommend regular speech evaluations, including pronunciation tests, for preschoolers with unilateral CND. Furthermore, it is crucial to educate parents or caregivers about the potential for pronunciation delays and encourage them to closely monitor their child’s speech development.

A notable strength of this study is that it represents, to the authors’ knowledge, the first analysis of pronunciation in children with unilateral CND, a topic not previously explored. Another strength lies in the presentation of specific numerical values and their comparison to the published norm data of typically developing children. However, the study does have limitations. As a retrospective medical record study, the data lacks homogeneity. Furthermore, the pronunciation score analysis only considered the accuracy of consonants, neglecting to assess vowels. The vocabulary score also has missing data, and the evaluation method lacks standardization. These limitations could potentially be addressed in a future prospective study using the same methodology.

In summary, preschoolers with unilateral CND tend to exhibit receptive vocabulary level that closely mirrors that of their typically developing peers but often struggle with poorer pronunciation which may not improve naturally, especially within their preschool years. Furthermore, one third of these children with significant delays in pronunciation accuracy also experience setbacks in vocabulary development. Consequently, it is crucial to conduct regular speech evaluations, including pronunciation tests, for preschoolers with unilateral CND. Parents or caregivers should be properly counseled and encouraged to closely monitor both pronunciation and vocabulary development in order to provide appropriate support and intervention as needed.

## Supporting information

S1 Table The demographic and clinical data of all subjects. (DOCX)
