# Supplementary material for: Comparing pronunciation challenges in South Korean preschoolers with unilateral single-sided deafness due to cochlear nerve deficiency to a norm-referenced standard
Source: PLoS One. 2024 Feb 23;19(2):e0297640. doi: 10.1371/journal.pone.0297640 (PMC10889857; doi:10.1371/journal.pone.0297640)
Supplement: S1 Table — (DOCX) [file pone.0297640.s001.docx]

**Supplementary Table 1.**

| **Patient No.** | **Sex** | **CND** | **CND side** | **Age in months** | **Pronunciation (z-score)** | **Pronunciation (result)** | **Vocabulary (z-score)** | **Vocabulary (result)** | **ABRT (CND side)** | **ABRT (contralateral)** | **PTA  (CND side)** | **PTA (contralateral)** |
| --- | --- | --- | --- | --- | --- | --- | --- | --- | --- | --- | --- | --- |
| 1 | M | H | R | 31 | -1.928 | Considered for AT | -1.708 | Mild delay | NR | 10 | 87.5 | 15.0 |
| 2 | M | A | L | 38 | -1.062 | Considered for AT | -1.208 | Mild delay | - | - | 105.0 | 15.0 |
| 3 | M | A | L | 31 | -3.281 | Recommended for AT | -2.217 | Severe delay | NR | 20 | 92.5 | 20.0 |
| 4 | M | A | L | 55 | -1.631 | Considered for AT | 1.086 | Above average | - | - | 87.5 | 12.5 |
| 5 | F | A | R | 47 | -4.855 | Recommended for AT | 1.826 | Above average | - | - | 90.0 | 11.3 |
| 6 | M | A | R | 42 | -1.132 | Considered for AT | -0.514 | Normal range | NR | 10 | 110.0 | 6.7 |
| 7 | F | A | R | 25 | -0.675 | Within normal limits | 2.374 | Above average | NR | 10 |  |  |
| 8 | F | A | L | 72 | -5.457 | Recommended for AT | -0.140 | Normal range | NR | 20 | 90.0 | 2.5 |
| 9 | M | A | L | 48 | -0.697 | Within normal limits | -0.120 | Normal range | NR | 20 | 82.5 | 7.5 |
| 10 | F | A | R | 63 | -0.844 | Within normal limits | 0.753 | Above average | - | - | 113.8 | 2.5 |
| 11 | M | H | R | 82 | -3.440 | Recommended for AT | 1.734 | Above average | NR | 20 | 116.3 | 10.0 |
| 12 | M | A | R | 29 | -1.026 | Considered for AT | 1.243 | Above average | NR | 20 | 101.3 | 10.0 |
| 13 | M | A | R | 62 | -4.792 | Recommended for AT | - | - | NR | 10 | 117.5 | 7.5 |
| 14 | M | A | R | 48 | -1.163 | Considered for AT | -0.476 | Normal range | 90 | 20 | 58.8 | 13.8 |
| 15 | M | A | R | 38 | -0.755 | Within normal limits | 0.179 | Above average | NR | 25 | 120.0 | 12.5 |
| 16 | M | A | R | 35 | -0.575 | Within normal limits | 0.599 | Above average | NR | 10 | 85.0 | 20.0 |
| 17 | F | A | L | 24 | -3.845 | Recommended for AT | -0.448 | Normal range | NR | 10 | 100.0 | 15.0 |
| 18 | M | H | L | 58 | -1.631 | Considered for AT | -0.643 | Normal range | - | - | 116.7 | 7.5 |
| 19 | F | A | L | 44 | -3.866 | Recommended for AT | -2.598 | Severe delay | NR | 10 | 92.5 | 23.8 |
| 20 | F | H | R | 24 | -4.710 | Recommended for AT | -1.018 | Mild delay | NR | 10 | 105.0 | 17.5 |
| 21 | F | A | R | 51 | -4.432 | Recommended for AT | - | - | NR | 10 | 105.0 | 6.3 |
| 22 | F | H | R | 41 | -7.823 | Recommended for AT | 1.913 | Above average | NR | 25 | 43.8 | 12.5 |
| 23 | M | H | L | 68 | -7.529 | Recommended for AT | 1.173 | Above average | - | - | 120.0 | 12.5 |
| 24 | M | H | L | 38 | -2.294 | Recommended for AT | 0.642 | Above average | NR | 10 | 110.0 | 11.7 |
| 25 | M | A | L | 32 | -3.056 | Recommended for AT | -1.688 | Mild delay | NR | 10 | 90.0 | 10.0 |

CND, cochlear nerve deficiency; ABRT, auditory brainstem response threshold; PTA, pure-tone average of the frequencies 0.5, 1.0, 2.0, 4.0 kHz; H, hypoplasia; A, aplasia; R, right; L, left; AT, articulation therapy; NR, no response; ‘-’ symbol in the test column means ‘not conducted’ or ‘no record’
